# Volatilome Analysis for Differentiating Terroir Expression: A Case Study of Three Wineries in a Limestone-Rich, Warm-Climate Region

**DOI:** 10.3390/molecules30142982

**Published:** 2025-07-16

**Authors:** José Miguel Fuentes-Espinosa, Raquel Muñoz-Castells, Jaime Moreno-García, Teresa García-Martínez, Juan Carlos Mauricio, Juan Moreno

**Affiliations:** Department of Agricultural Chemistry, Edaphology and Microbiology, Marie Curie (C3) and Severo Ochoa (C6) Buildings, Agrifood Campus of International Excellence CeiA3, University of Córdoba, Ctra. N-IV-A, km 396, 14014 Córdoba, Spain; b42fuesj@uco.es (J.M.F.-E.); b52mucar@uco.es (R.M.-C.); b62mogaj@uco.es (J.M.-G.); mi2gamam@uco.es (T.G.-M.); mi1gamaj@uco.es (J.C.M.)

**Keywords:** wine, terroir, vintage, volatile compounds, chemometric differentiation

## Abstract

This study investigated young white wines produced during the 2021 and 2022 vintages from Pedro Ximénez grapes cultivated in three different terroirs within a high-quality production zone. The general oenological parameters were significantly influenced by vintage and terroir (*p* ≤ 0.001), with ethanol and reducing sugars specifically affected by the terroir and its interaction with the vintage. Multivariate analysis of major and minor volatile compounds enabled the characterization of terroir-specific volatile profiles. However, principal component analysis (PCA) grouped samples by vintage rather than terroir. Ethyl esters of medium- and long-chain fatty acids and certain acetates of higher alcohols were the most discriminant volatiles and were proposed as key compounds for differentiating wines by terroir and vintage. These findings underscore the influence of the terroir on the volatilome and support its relevance in defining wine typicity and quality.

## 1. Introduction

For centuries, winemaking has been considered both an art and a science, influenced by tradition, geography, and innovation. Wine consumers and experts are often amazed at how the same grape variety can produce very different wines depending on where it is grown and the techniques used to produce it. This phenomenon is attributed to the “terroir”, a concept that encompasses the environmental, geological, and human factors that influence wine quality. In a global market that demands consistency and authenticity in wine production, the challenge lies in balancing the control of winemaking processes with the preservation of regional typicity. Understanding the chemical and microbial factors that define a wine’s identity is important for both producers and consumers who seek high-quality wines that express their origin. According to the Organisation Internationale de la Vigne et du Vin (OIV), terroir is defined as “an area in which collective knowledge of the interactions between the identifiable physical and biological environment and applied vitivinicultural practices develops, providing distinctive characteristics to the products” [1].

Terroir integrates multiple variables, including soil composition, topography, climate, grapevine genetics, grape microbiota composition, and viticultural and enological practices [2,3]. These factors influence vine physiology and grape biochemistry, ultimately determining the chemical and sensory characteristics of the wine [4,5]. While climate and grape variety are often emphasized, soil and its biological aspect are gaining recognition as critical determinants of terroir expression [6]. In this regard, soil provides the physical and biological support necessary for grapevine development. Its texture, structure, mineral composition, drainage capacity, and organic matter content directly influence vine vigor, root depth, water uptake, and nutrient availability [3,7,8,9]. For instance, gravel-rich soils improve drainage and restrict vegetative growth, leading to smaller berries with concentrated flavors. In contrast, clay-rich soils retain water and nutrients, which often increases yield but reduces flavor intensity [10].

Beyond its physical properties, the soil is a living system that hosts diverse microbial communities. These communities interact with grapevine roots and play an important role in nutrient cycling, hormone signaling, and even disease resistance [9,11,12]. The microbial composition and functionality of soil microbiomes have been shown to influence grape metabolome and fermentative outcomes. This suggest that microbial profiles are terroir-specific and correlate with the chemical fingerprint of wines [13,14,15,16].

Grape varieties bring genetic potential to terroir expression. However, their sensory characteristics, such as aroma, flavor, and phenolic content, are highly dependent on environmental interactions [8,17,18]. For example, Cabernet-Sauvignon or Pinot Noir show different characteristics depending on soil type, altitude, and regional climate, producing markedly different wines in Burgundy, Oregon, or New Zealand [19,20]. The success of any grape variety depends on its ability to adapt to specific environmental conditions. Site-specific selection, based on knowledge of soil and climate, is essential to optimize vine balance and wine expression [21,22]. The grape variety also influences the assembly of microbial communities on the berry surface, which in turn affects the fermentation of the grape must and the volatilome development [11,23].

The volatilome is of a set of volatile organic compounds (VOCs) that define the aroma profiles of grapes and wines. These volatiles mainly include esters, alcohols, acids, aldehydes, and terpenes, which come from grape precursors, yeast metabolism during fermentation, and aging processes [17,18,19,20,21,22,23].

An increasing number of studies show that terroir has a measurable effect on the volatilome. Differences in soil composition, microbial populations, and vine stress levels lead to different VOC profiles, which can serve as chemical markers of geographic origin [24,25,26,27]. For instance, wines from volcanic soils exhibit sulfur- and mineral-based odor notes, while limestone-based soils enhance floral and citrus aromas [9,28].

Advanced metabolomics techniques, such as Gas Chromatography–Mass Dpectrometry (GC-MS) and untargeted profiling, now enable high-resolution analysis of the grape and wine volatilome, helping researchers link aromatic profiles to terroir-specific conditions [18,29,30]. These techniques reveal that the volatilome is shaped by grape genotype, microbial dynamics, fermentation conditions, and the vineyard soil microbiota [4,7,24,31].

The interaction between soil, grape variety, and microbial and chemical processes results in a wine’s volatilome, which is a strong indicator of terroir expression [14,25]. Understanding these relationships allows viticulturists to manage soils more effectively and select the appropriate varieties, while providing winemakers with tools to enhance aroma development during vinification.

The concept of microbial terroir emphasizes the importance of an integrative approach to viticulture and enology. Multi-omics techniques, which combine soil microbiology, grape biochemistry, and volatile compound profiling, offer a scientifically precise way of understanding terroir [16,25,26,27,28,29]. This understanding may contribute to preserving the cultural identity of wines and drive innovation in vineyard management and wine production.

A key challenge in modern oenology is the objective characterization of wine typicity within a given terroir. Although traditional wine regions emphasize the unique sensory attributes of their wines, scientific validation of these differences is limited. All of the aforementioned factors influence terroir effects and play an important role in wine sensory profiles; however, a standardized approach to quantifying these influences is still lacking. Currently, several studies focus on grape variety and winemaking techniques, while the role of terroir-specific volatiles in wine differentiation has not been thoroughly investigated [2,5,31,32,33].

This study aims to address the lack of knowledge by applying GC-MS analytical techniques and chemometric analysis to characterize the volatilome of wines produced from the same grape variety grown in three terroirs with the same soil type and climatic conditions and similar viticultural and enological practices. Building on previous work [34], this study is conducted in a subzone of the Protected Designation of Origin (PDO) Montilla-Moriles in Southern Spain. Using multivariate statistical techniques, we aim to identify the key volatile compounds or group of compounds that define the typicity of each terroir’s wine. This analytical approach will help to scientifically distinguish wines by terroir and deepen our understanding of the chemical markers behind sensory perception and authenticity in traditional high-quality winemaking regions.

## 2. Results and Discussion

### 2.1. Oenological Parameters

The results of the general compositional analysis of Pedro Ximénez wines from the three terroirs, located in the highest quality zone of Montilla-Moriles PDO in the 2021 and 2022 vintages, are given Table 1. This table shows the average contents, standard deviations, and homogeneous groups (HGs) at the *p* ≤ 0.05 significance level of these six wines.

In both vintages, the ethanol, sugar content, and density have typical values for this production zone and define these wines as being dry types. Reducing sugars exhibit four HGs, whereas both ethanol content and density each show three HGs. Volatile acidity, total acidity, absorbances at 420 nm, and the Total Polyphenol Index each present six HGs, indicating a strong association with the winery, terroir, and vintage year.

The levels of malic and lactic acid are grouped into five HGs. The two acids are the substrate and the product of malolactic fermentation, respectively, which depend on the winemaking practices of each cellar. Lactic acid bacteria are responsible for the conversion of malic acid into lactic acid, and it is usually induced by winemakers to obtain high-quality red wines, but it is only desirable in the elaboration of these white wine types. Malolactic fermentation is very dependent on the ethanol content and the pH values, both of which depend on the criteria of each winery. Consequently, both vintage and terroir seem to contribute to the observed variation. The HGs obtained for each parameter summarize, at the *p* ≤ 0.05 level, the combined effects of the vineyard environment and the oenological practices of each winery on the typology and general characteristics of the wines from this subzone. Also, a two-factor analysis of variance (vintage and terroir) and an analysis of their interactions, performed for each parameter, shows that all of them, except for ethanol and reducing sugars, are dependent at a *p* ≤ 0.01 level on both factors and interactions (Supplementary Table S1).

The values obtained for the parameters analyzed are within the range established by the legislation of the Montilla-Moriles PDO [35] for the production of Fino-type wines, particularly the ethanol content, reducing sugars, and total and volatile acidity. Similarly, the aforementioned parameters, together with the others, are comparable to those shown in a study carried out by the same authors on young industrial wines of the same grape variety but from a different production subzone [34] and on young wines obtained from spontaneous fermentations at the laboratory scale with musts of the same variety and vintage [36].

### 2.2. Major Volatile Compounds and Polyols

The contents of this group of VOCs in the six wines (three terroirs and two vintages), all of them analyzed by direct injection in a GC, are shown in Table 2. The contents are expressed by the means and standard deviations (in mg L^−1^) of the three analytical replicates.

The aroma of wine is primarily driven by the secondary metabolism of yeast during alcoholic fermentation. Volatile metabolites produced during this process are classified as major volatile compounds if their concentration is ≥10 mg/L. These compounds include carbonyl compounds, higher alcohols, and ethyl esters of acetic, succinic, and lactic acids. Yeasts also synthesize low-volatility secondary metabolites, such as the polyols 2,3-butanediol (*levo* and *meso* forms) and glycerol. The production of these metabolites is influenced by multiple factors, including fermentable sugar and assimilable nitrogen content, fermentation temperature, yeast strain, and yeast-derived nutrients [37]. This study quantifies three carbonyl compounds, methanol, five higher alcohols, three ethyl esters of short-chain organic acids, and three polyols.

Acetaldehyde and 1,1-diethoxyethane show four HGs and acetoin shows three HGs at the *p* ≤ 0.05 significance level. All of these compounds have higher concentrations in the 2021 wines. These compounds are related to the metabolic activity of *Saccharomyces* yeasts, which grow spontaneously in young wines from this PDO after alcoholic fermentation. These yeasts develop a biofilm on the wine’s surface and are responsible for the biological aging process. This special type of wine aging is traditionally carried out over several years in the PDO studied, as well as in others such as Jerez-Xeres-Sherry in Andalusia (southern Spain) [38]. However, the wines in this study did not undergo this process.

Higher alcohols contain more than two carbon atoms. They are synthesized via the Ehrlich pathway from the keto acid pool. While these compounds enhance the aroma and complexity of wine, concentrations exceeding 400 mg L^−1^ can negatively impact its sensory quality [39]. Of the five quantified higher alcohols, 2-phenylethanol, 1-propanol, and isobutanol each exhibit five HGs, while 3-methyl-1-butanol shows four HGs, and 2-methyl-1-butanol shows three HGs. Methanol also shows three HGs. Among the higher alcohols, only 2-phenylethanol has a pleasant, rose-like aroma and occurs at higher levels in LS wines, whereas isobutanol reaches its highest concentrations in CN wines. LS wines also have the highest content of 3-methyl-1-butanol, which has a chemical solvent odor descriptor.

The ethyl esters of acetic, lactic, and succinic acids are the most abundant ester family in wine. Ethyl acetate and diethyl succinate have five HGs, while ethyl lactate has four HGs. The latter two have higher concentrations in the 2021 wines. However, ethyl acetate has higher concentrations in the 2022 wines. Values higher than 60 mg/L of ethyl acetate can result in varnish-like aromas, whereas lower concentrations provide pleasant, fruity aromas, such as pineapple. Ethyl lactate exhibits sweet, lactic acid, and yogurt aromas, while diethyl succinate has an overripe melon odor [40,41].

Finally, the semi-volatile compound 2,3-butanediol (in its *levo* and *meso* forms) and the non-volatile compound glycerol have two, three, and four HGs, respectively. These compounds generally show a higher dependence on vintage year, with higher concentrations in the 2022 wines.

### 2.3. Minor Volatile Compounds—Screening of Chemical Families for Best Differentiation of Terroir Effects

Table 3 lists the 46 minor volatile compounds quantified in the wines, grouped into eight chemical families. All of them have contents lower than 10 mg L^−1^ at parts per billion or microg L^−1^ levels, except γ-butyrolactone. The ester family is qualitatively the most abundant family with 21 compounds, of which 6 are acetates of higher alcohols, 12 are ethyl esters of medium- and long-carbon-chain acids, and 3 are other esters of phenethyl alcohol. All of them have great influence on wine aroma because of their low-odor-perception thresholds and pleasant fruity aroma [39,42].

Only 2-phenylethyl acetate and phenethyl hexanoate have two HGs; butyl acetate, octyl acetate, and phenethyl benzoate have three HGs; and isoamyl acetate and ethyl octanoate have five HGs, representing the highest levels in the 2022 vintage for all terroirs. The remaining 14 esters have four HGs at the *p* ≤ 0.05 significance level. It is also relevant to note that the ethyl esters of the C6, C8, C10, C12, C14, and C16 acids also show a higher concentration in the 2022 vintage. The content of medium- and long-chain fatty acids and their ethyl esters is influenced by several factors. The most important factor is the stress that different yeast species undergo during alcoholic fermentation [43]. Since the dominant yeast species in this process are associated with the terroir [36], it is very likely that the contents found in this study are dependent on the effect of the specific yeasts of each terroir.

Regarding the higher alcohol family, all five quantified in this work show three HGs and only hexanol, 2-ethyl-1-hexanol, and 1-dodecanol show quantifiable amounts in all wines. Hexanol is highlighted among the remaining due to its higher content in wines from the 2022 vintage in the three terroirs. This feature is also observed for the 2-methoxy-4-vinylphenol in the phenol family that shows five HGs. Hexanol content in wines comes from the lipoxygenase activity from the fatty acids of grapes and the volatile phenols which also come from the grapes or from the metabolism of some non-*Saccharomyces* yeasts that are present in the grapes of specific terroirs [34,44].

Three lactones are quantified within this chemical family, with γ-butyrolactone being the most important in terms of its content. It is excreted to the wine by *Saccharomyces cerevisiae* during their fermentative metabolism and shows four HGs, as does the β-damascenone. In contrast, γ-nonalactone has five HGs. All lactones show higher concentrations in the 2022 wines. As potent odorants, their levels are associated with grape-derived precursors and the aging of wine in oak barrels [45].

The carbonyl compounds consist of seven aldehydes and one ketone. Only furfural (furaldehyde) and hexanal, both with three HGs, and decanal (two HGs) have quantifiable amounts in all wines. Hexanal, octanal, and decanal are derived from the enzymatic hydrolysis of long-chain fatty acids, as is the hexanol-1 described previously. The high furfural contents of these wines are explained by its formation via the Maillard reaction [46] from the sugars of grapes, which are partially dehydrated on the vine a few days before the harvest. These changes are traditionally observed in the warm grape-growing zone where the wineries are located and are a consequence of the high temperatures reached (higher 40 °C) during the months of August and September, when grapes are ripened and harvested.

The terpenes Z-citral and Z-nerolidol and the terpene derivatives geranyl acetate, E-methyldihydrojasmonate, and Z-geranyl acetone constitute the last chemical family of volatiles quantified. Only derivatives of geraniol were quantified in all the wine samples, with its acetate being the sole compound among the 46 quantified to display six HGs, whereas the other terpenes showed only two or three HGs. These low contents are in accordance with the classification of Pedro Ximénez grape as a neutral variety, <0.3 mg L^−1^, which results in a weak contribution to the wine aroma [47].

To identify the compounds significantly related to terroir and vintage effects, a multifactorial analysis of variance was performed on all quantified compounds (general parameters, major volatiles, polyols, and minor volatiles). The results of this analysis show that practically all of them are significantly dependent on terroir, vintage, and their interaction at the *p* ≤ 0.01 level. Only the general parameters ethanol and reducing sugars; the major volatiles methanol, isobutanol, and 2-methyl-1-butanol; and the minor volatiles 2-phenylethyl acetate, ethyl butyrate, 2-furan methanol, dodecanol, benzaldehyde, decanal, and (E)-geranylacetone are not dependent on some of these factors at the *p* ≤ 0.01 level. (Table S1 in the Supplementary Material).

In summary, these results do not allow for the identification of a single compound that would be considered significant as a key compound to differentiate the six wines by terroir or vintage.

### 2.4. Selection of a Set of Volatile Compounds and Chemical Families for a Better and Easier Differentiation Among Terroirs

There are different strategies for handling large amounts of data. Qualitatively, heat maps stand out, followed by multiple variable analysis (MVA), which easily visualizes differences between samples. Quantitatively, PCA is the most common statistical treatment.

Heat maps are used to identify biomarkers related to terroir, establish relationships with sensory characteristics, and classify wines according to the yeasts used in their production [48,49,50]. The heat map generated with the contents of 46 minor and 14 major volatiles (Figure S1 in Supplementary Material) shows the differences between wines according to terroir and vintage, making it easy to compare them. However, the results do not allow for the optimal grouping of wines from the LB winery and the 2021 and 2022 vintages, since their positions are interchanged.

**Figure 1:**
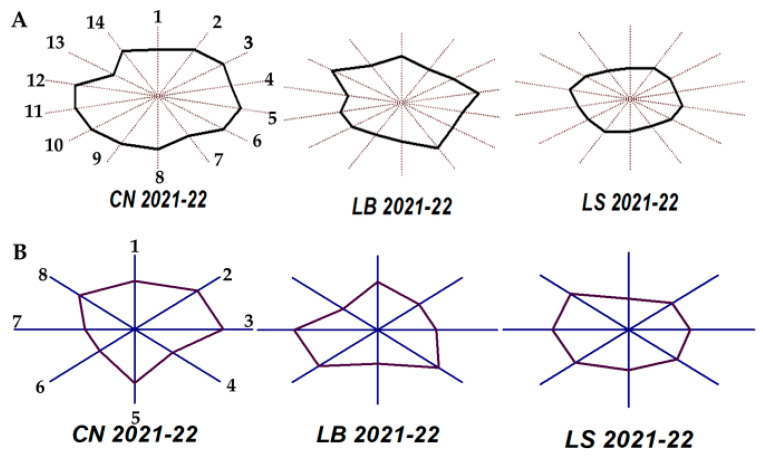
Fingerprints obtained by the multivariate analysis of wines from three terroirs of “Sierra de Montilla” obtained in the 2021 and 2022 vintages. (**A**): Average of the major volatile compounds and polyols. The rays and numbers in the polygons correspond to the following: 1: acetaldehyde; 2: ethyl acetate; 3: 1,1-Diethoxiethane; 4: methanol; 5: 1-Propanol; 6: isobutanol; 7: isoamyl alcohols; 8: acetoin; 9: ethyl lactate; 10: 2,3-Butanediol (*levo*); 11: 2,3-Butanediol (*meso*); 12: diethyl succinate; 13: 2-Phenylethanol; 14: glycerol. (**B**): Average of the minor volatile compounds from the 2021 and 2022 vintages grouped in eight chemical families. The rays and numbers in the polygons correspond to the following: 1: acetates; 2: ethyl esters; 3: other esters; 4: higher alcohols; 5: volatile phenols; 6: lactones; 7: carbonyl compounds; 8: terpenes and derivatives. CN: Cañada Navarro; LB: Los borbones; LS: Lagar Saavedra.

With the aim of easily visualizing the influence of terroir, an MVA was performed on the average values of the major volatiles and also on the chemical families of the minor volatiles quantified in two vintages. Figure 1 shows the results obtained using the so-called sun-ray plot, which is very useful for identifying differences and similarities between observed cases when the number of measured variables is high.

The polygon obtained by joining the values of each quantitative variable in each sample represents its fingerprint. The size of the polygon in each direction or ray is scaled according to the value of each variable for the winery associated with a terroir. Wineries with similar characteristics will have a similar size and shape. When the option means and sigma scales were chosen for the MVA, the origin of the rays represented the mean of each variable minus three times its deviation, and the extreme corresponded to the mean plus three times its deviation.

According to Figure 1A, the LS winery has the most regular polygon and LB the most irregular. The LB winery has the highest levels of 2-phenylethanol. CN, although it has the lowest level of this higher alcohol, it shows higher contents of the remaining compounds than the other wineries. Regarding the minor volatile families, winery LS has the most regular polygon considering the two vintages. CN shows higher levels of ethyl and other esters and phenols, while LB shows the highest values of acetates, alcohols with six or more carbon atoms, lactones, and carbonyl compounds. CN and LS have high and similar values of terpenoid compounds. These fingerprints facilitate the authentication of wines by origin and/or vintage, thus protecting producers and consumers against possible counterfeiting or fraud.

The differences in the wine volatile profiles of the wineries between vintages can be attributed to their microbiota, which influences the distribution of subregional and varietal communities in specific terroirs and, consequently, the profile of the wine [51]. In this regard, the three wineries presented 174 yeast isolates in the spontaneous fermentation of their grape musts under controlled laboratory conditions. The CN winery showed a balanced percentage of *Saccharomyces* (52%) and non-*Saccharomyces* (48%) yeasts, while the LB and LS wineries showed a higher percentage of non-*Saccharomyces* yeasts, reaching 55% and 60%, respectively [36].

Some researchers suggest that vintage is more important than terroir (geographical location) in determining yeast biodiversity in grapes [47,52]. This finding explains the differences observed in the wines from the three wineries in vintages 2021 and 2022. However, the results also show similar groupings of wines by terroir in the two vintages, suggesting there is always also a strong influence of terroir on their specific microbiota under favorable microclimatic conditions. In this regard, it is observed that terroir has a strong influence on the concentrations of some families of aroma compounds in the resulting wines [53].

With this in mind, and with the aim of identifying the combination of volatile compounds that contributes the most variability to the wine differentiation, a PCA was performed on the major volatiles and polyols and on the 46 minor volatiles. Also, a PCA was performed on the content of terpenoid compounds and 21 minor esters, since some components of these families of compounds have the largest number of homogeneous groups and the heat map shows greater variability between samples. These families of compounds were chosen for ease of analysis and because some of them have been selected by other authors as being influenced by terroir effects [7,53].

**Figure 2:**
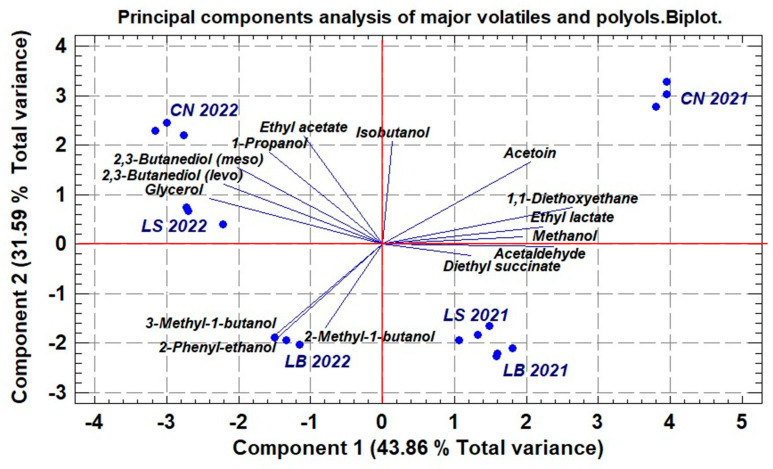
Principal component analysis of major volatiles and polyols quantified by direct injection of wines from three terroirs of “Sierra de Montilla” obtained in 2021 and 2022 vintages. CN: Cañada Navarro; LB: Los borbones; LS: Lagar Saavedra.

The results of the PCA performed on the contents of the main volatiles and polyols are shown in Figure 2. This analysis provides an approach to explore and visualize a preliminary classification of wines using a limited set of compounds quantified in a single step by the direct injection of the wine into the GC, according to the method used in this study.

This biplot shows PC1 and PC2 as the orthogonal axes, explaining 75.45% of the total variance. The 2021 wines are on the right, and the 2022 wines are on the left. PC1 groups the wines by vintage, and PC2 by terroir. The volatiles influencing PC1 with positive coefficients are the three carbonyl compounds, methanol, ethyl lactate, and diethyl succinate, while glycerol, butanediol (*levo* and *meso* forms), and 2-methyl-1-butanol and 3-methyl-1-butanol contribute negative coefficients to the wines. The 2022 wines are more separated by terroir than those of 2021. The most differentiated terroir in 2021 is CN, while LS and LB are very close in their scores in PC1 and somewhat further apart in PC2.

Component 2, explaining 31.59% of the variance, is driven by positive contributions from isobutanol, ethyl acetate, and 1-propanol and negative loads of 2-methylbutanol, 3-methylbutanol, and 2-phenylethanol (Table S2, Supplementary Material). These results suggest that higher alcohols play a key role in the separation of wine samples by terroir and carbonylic compounds jointly with glycerol and the ethyl esters of lactic and succinic acids separate wine samples by vintage.

To find a group or family of volatiles that would allow a more significant and objective classification of wines with respect to their terroir, a PCA was performed on the 46 quantified volatiles. This analysis resulted in three components explaining 77.595% of the total variance (Supplementary Material Figure S2 and Table S3). PC1 and PC2 explain 39.853 and 25.082%, respectively, of the total variance and PC3 12.660%. The scores of wines in both PC1 and PC2 are plotted in Supplementary Figure S2 and the contribution (loadings) of each compound to the PCs are listed in Table S3. These loadings allow us to identify the most important volatiles influencing each PC. In this regard, 12 esters are the main volatiles contributing to PC1; 9 to the PC2, from which 7 are different those influencing PC1; and lastly, 3 esters (2 different) influence the PC3. In this way, all 21 quantified esters have significant loadings in the three PCs, suggesting an important contribution of this chemical family to the classification of wines. Also, the groups of terpenes and their derivates contribute with important loads to the three considered PCs. The plot of wine scores in PC1 and PC2 shows that wines of the 2021 vintage are located to the right and the 2022 wines to the left, but the separation among terroirs in both vintages is not good. Also, the scores of PC1 and PC2 obtained with the seven terpenic compounds do not allow for a good separation of wines by terroir, although they do show a separation by vintage (Supplementary Figure S2B). The loads of each terpenoid in the three PCs are listed in Supplementary Material Table S4.

**Figure 3:**
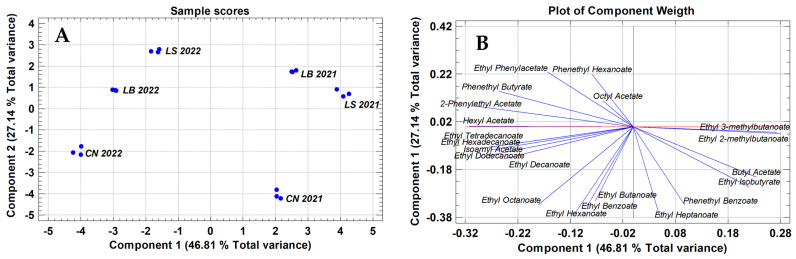
Principal component analysis of 21 esters differentiating the vintage and terroir effects of wines from the three terroirs of “Sierra de Montilla” and the 2021 and 2022 vintages. (**A**): Sample scores of wines in the first two principal components. (**B**): Contribution of each ester to the two first components. Identification of wineries/terroirs: CN: Cañada Navarro; LB: Los borbones; LS: Lagar Saavedra.

Lastly, the PCA carried out on the 21 minority esters allows us to obtain the best separation of wines by terroir, as is shown in Figure 3A. All wines from vintage 2021 are located on the right and those from 2022 vintage are on the left of this plot. This distribution is driven by the sample scores of the wines in PC1 and PC2, which explain 46.81 and 27.41, respectively, of the total variance supported by these esters. PC1 is influenced by ethyl esters of C10, C12, C14, and C16 organic acids, jointly with negative loads from the acetates of isoamyl, hexyl, and 2-phenylethanol alcohols and the phenethyl butyrate. By contrast, the ethyl esters of isobutanoic, 2-methylbutanoic, and 3-methylbutanoic acids and butylacetate have positive loads.

The sample scores for PC1 group the wines by the year of harvest, and therefore may be representative of the vintage effects. The most important esters influencing PC2 are the ethyl esters of iso-butyric, butyric, benzoic, C6, C7, and C8 organic acids and the acetate of butyl alcohol, all of which have negative loads, while ethylphenyl acetate, phenethyl butyrate, and phenethyl hexanoate show positive loads (Supplementary Material Table S5). The scores of wines in PC2 show the best separation of wines by terroir, suggesting a relationship between the contents of these esters and this factor.

The relationships obtained between esters and terroir underline in an objective way the recognized sensorial properties of their wines, which are described to have a more fruity and sweet aromatic profile than those from other subzones in the same PDO. The results highlight the complex interplay between terroir and vintage year in the volatilome profile of wines from different terroirs located in the same high-quality subzone of a wine-growing production area.

The results of this study demonstrate that the analysis of volatile compounds (major and minor) in wine, using advanced statistical tools such as PCA, can effectively authenticate wines as representative products of specific terroirs. Further research is essential to deepen our understanding of the complex relationship between terroir and wine quality. Such studies should aim to establish objective markers based on key volatile compounds to ensure the typicality and quality of wines from traditional production areas. This authentication of geographical origin involves the bodies that issue the certifications that guarantee the quality and origin of wines and those that regulate the norms and laws governing wine production and labeling.

## 3. Materials and Methods

### 3.1. Location of Terroirs and Wineries

This work was conducted in three wineries widely recognized for the high quality of their products. These wineries and their respective vineyards are located in a subzone known for its gently sloping terrain and altitudes ranging from 400 to 600 m, and the work was conducted in the 2021 and 2022 vintages. This subzone, called “Sierra de Montilla,” is classified as a premium wine-growing area within the Montilla-Moriles Protected Designation of Origin (PDO) in Andalusia, southern Spain [35].

According to the Köppen classification system, this geographical region has a Csa climate, characterized by hot, dry summers and mild, wet winters. This climate type is identified by average monthly temperatures above 10 °C for at least four months, with the warmest month exceeding 22 °C. Precipitation is abundant in winter and scarce in summer.

Climatic data from the nearest meteorological station of the state meteorological agency (AEMET) for the years 2021 and 2022 showed average maximum temperatures of 24.6 °C and 25.8 °C with absolute maximums of 46.1 °C and 43.1 °C. The average minimum temperatures were 11.5 °C and 12.2 °C, with absolute minimums of −3.5 °C and 0.3 °C. Lastly, the precipitation for these years was 330 and 427 mm, respectively.

The wineries included in this study are located at the following GPS coordinates: Cañada Navarro (CN): 37°32′28.96800″ N, 4°33′25.52400″ W; Lagar Saavedra (LS): 37°32′55.44888″ N, 4°32′28.64400″ W; and Los borbones (LB): 37°33′27.99720″ N, 4°33′35.38800″ W. Their respective vineyards grow only Pedro Ximénez grapes, which are well-adapted to the soil and climate of this region. Cañada Navarro uses a double-cordon pruning system combined with a vertical trellis system for its vines, while the remaining vineyards use a traditional head pruning system [54]. Each winery uses only the grapes grown on its property and in nearby vineyards for wine production.

This premium subzone is defined by its calcareous “albariza” soils, of which the parent materials are mainly marls and limestones, with high calcium carbonate (>700 g kg^−1^) and low organic matter, non-crystalline Fe oxides, and available Fe. Also, vines cultivated in this environment typically exhibit an increased leaf surface area exposed to solar radiation [54].

### 3.2. Grape Variety and Wine Sampling

The grape variety used across all three vineyards is Pedro Ximénez. Manual harvesting was carried out during two consecutive vintages (2021 and 2022) in September once the grapes reached a sugar concentration of at least 250 g L^−1^—sufficient to yield wines with a potential alcohol by volume (ABV) of 14.5% or higher. The vinification processes were conducted on an industrial scale at each winery using traditional practices in stainless steel containers with a capacity of 50–80 hL, and according to the rules of the CRDOP “Montilla-Moriles” [35]. These rules included gentle pneumatic pressing to extract the must (70 L per 100 kg of grapes), pH adjustment with tartaric acid to a recommended pH value around 3.3, and a settling period of 24 to 48 h. Fermentation took place at a controlled temperature of 18 ± 2 °C with indigenous yeasts, using the traditional “pied de cuve” method, which allowed the selection of the yeasts that were naturally present in the microbiome of each vineyard. No yeast starter cultures were added. The “pied de cuve” procedure ensures that suitable yeast strains linked to the terroir are acclimated first and then used as an inoculum for fermentation. Additional must fractions were incrementally added during the yeast’s exponential growth phase. This fed-batch strategy enhances yeast performance, prevents nutrient depletion, and maintains fermentation activity until the containers reach capacity. Under these conditions, fermentation typically lasts about one month, ensuring the production of dry white wines with a higher alcohol content. After a one month spontaneous stabilization period, three samples of young wines were collected in each winery and vintage for analysis. The results obtained for each year and winery were averaged and shown in the tables and figures.

### 3.3. Oenological Parameter Analysis

Standard parameters for preliminary wine profiling—including ethanol content, titratable and volatile acidity, pH, and residual sugars—were measured using the analytical methods recommended by the International Organisation of Vine and Wine (OIV) [55]. Concentrations of lactic and malic acids were determined using the reflectometric methodology with a Reflectoquant™ system (Merck^®^, Darmstadt, Germany). Absorbance measurements at 280 and 420 nm were obtained with a Cary 60 UV-Vis spectrophotometer (Agilent Technologies, Santa Clara, CA, USA). The Total Polyphenol Index (TPI) was measured using the method described by Ribéreau-Gayon et al. (2000) [56].

### 3.4. Quantification of Major and Minor Volatile Compounds

Volatile compounds in wine samples were analyzed according to the previously published methods used in our laboratory [57,58]. Briefly, different methods were used to determine each of the main groups of volatiles: major compounds and polyols, with contents ≥10 mg L^−1^, and minor compounds (contents below 10 mg L^−1^). The first group of compounds was quantified using an Agilent 6890 GC with FID and a CP-WAX 57 CB capillary column via direct wine injection. Samples were prepared by adding 1 mL of the internal standard (4-methyl-2-pentanol), along with 0.2 g of CaCO_3_, to 10 mL of the sample. This was then subjected to ultrasonic agitation and centrifugation, after which the supernatant was directly injected. Target compounds included methanol, higher alcohols, acetaldehyde, acetoin, ethyl acetate, glycerol, and both *meso*- and *levo*-2,3-butanediol. Calibration curves based on standard solutions were used for quantification.

Minor volatile compounds were identified and quantified by Stir Bar Sorptive Extraction followed by Thermal Desorption and Gas Chromatography–Mass Spectrometry (SBSE-TD-GC-MS) methods. This was conducted using an Agilent 7890A GC coupled with a 5975C mass selective detector and a Gerstel MPS autosampler (Mülheim an der Ruhr, Germany). The system was operated using Chemstation v. 02.02.143 and Maestro software v. 1.3 from ChemStation International, Inc., Dayton, OH, USA, and Gerstel Mülheim an der Ruhr, Germany, respectively. Extraction of volatiles was performed using PDMS-coated stir bars (Twisters^®^_,_ Gerstel, Mülheim an der Ruhr, Germany), followed by desorption in a Thermal Desorption Unit. Then, a 10 mL vial was filled with 1 mL of wine, 0.1 mL of the internal standard (0.4116 g/L of hexyl butyrate in absolute ethanol), and 8.9 mL of a buffered ethanol solution (12% *v*/*v*, pH 3.5) containing 2.6 g/L of tartaric acid and 2.2 g/L of potassium bitartrate. Then, one Twister^®^ stir bar was added, and the mixture was stirred at 1200 rpm and 20 °C for 120 min using a Variomag multipoint magnetic stirrer (Thermo Fisher Scientific, Waltham, MA, USA). After this time, the stir bar was rinsed, dried, and transferred to the TDU via the MPS for volatile desorption and GC-MS analysis using an HP-5MS fused silica column (60 m, 0.25 mm, 0.25 µm) from Agilent. Quantification was based on calibration tables obtained from standard solutions made with pure chemicals (Sigma-Aldrich, St. Louis, MO, USA and Merck) using the methodology previously described in articles from our research group [57,58]. Identification was carried out by comparing spectra against the NIST08 and Wiley7 libraries, and further validated using the mass spectra of pure compounds.

### 3.5. Statistical Analysis

Statistical analyses were performed on data matrices that included general oenological parameters, as well as major and minor volatile compounds, using Statgraphics Centurion (v. 16.1.11). A multivariate analysis of variance (MANOVA) was conducted to evaluate the effects of terroir, vintage, and their interaction. To identify homogeneous groups, analyses of variance (ANOVAs) with multiple range tests and F-tests were applied. Additionally, multivariate analysis (MVA) was used to identify significant differences among wines from the three terroirs across two vintages, and a heat map was generated using the MetaboAnalyst web platform [59]. Principal component analysis (PCA) was also conducted to summarize and interpret patterns in the concentrations of volatile compounds, polyols, and minor volatiles.

## 4. Conclusions

This study analyzed young white wines made from Pedro Ximénez grapes grown in three terroirs located in the top-quality subzone of a PDO, focusing on the 2021 and 2022 vintages. The primary aim was to assess how the terroir influences the wines’ volatile compound profiles.

Analysis of the general oenological parameters showed statistically significant differences (*p* ≤ 0.001) based on both vintage and terroir for all of them, except for ethanol and reducing sugar levels, which were specifically influenced by terroir and its interaction with vintage. MVA of both major and minor volatile compounds from the two vintages revealed the different chemical fingerprints associated with each terroir.

PCA grouped the wines according to vintage but did not differentiate them by terroir when using either the 14 major volatile compounds and polyols or the matrix of 46 quantified minor volatiles, including terpenoids.

Ethyl esters of short-, medium-, and long-chain fatty acids, along with certain acetates of higher alcohols, demonstrated the strongest ability to distinguish between the effects of terroir and vintage on the wines’ volatile profiles. These compounds likely contribute to the traditionally described sensory differences of the wines of the studied terroirs.

The findings underscore the impact of terroir—even within the same viticultural region—on the volatile composition of wines, supporting its role in defining product identity and quality. The results also point to the need for further research to better understand and objectively characterize wine typicity through key compound profiles.

## Figures and Tables

**Table 1:** Mean values and standard deviations of the general parameters of wines from three wineries located in the top-quality zone “Sierra Montilla” (Protected Designation of Origin “Montilla-Moriles”) during the 2021 and 2022 vintages. The different letters ^a, b, c, d, e,^ and ^f^ in the same row indicate homogeneous groups (HGs) at the *p* ≤ 0.05 significance level. Wines are from the following: CN = Cañada Navarro; LS = Lagar Saavedra; LB = Los borbones. TPI: Total Polyphenol Index, as the absorbance unit.

Parameters	CN2021	CN2022	LS2021	LS2022	LB2021	LB2022	HGs
Ethanol (% *V*/*V*)	14.0 ± 0.0 ^a^	14.5 ± 0.0 ^b^	15.0 ± 0.1 ^c^	14.5 ± 0.0 ^b^	15.0 ± 0.1 ^c^	15.0 ± 0.1 ^c^	3
pH	3.43 ± 0.01 ^d^	3.40 ± 0.00 ^c^	3.40 ± 0.02 ^c^	3.24 ± 0.01 ^a^	3.35 ± 0.01 ^b^	3.47 ± 0.01 ^e^	5
Volatile acidity (g L^−1^)	0.39 ± 0.00 ^f^	0.36 ± 0.00 ^e^	0.33 ± 0.00 ^d^	0.24 ± 0.00 ^b^	0.26 ± 0.02 ^c^	0.18 ± 0.00 ^a^	6
Total acidity (g L^−1^)	4.31 ± 0.00 ^b^	5.55 ± 0.00 ^f^	4.24 ± 0.00 ^a^	4.95 ± 0.00 ^e^	4.77 ± 0.03 ^d^	4.39 ± 0.00 ^c^	6
Malic acid (g L^−1^)	0.27 ± 0.00 ^b^	0.53 ± 0.03 ^c^	0.01 ± 0.00 ^a^	0.90 ± 0.01 ^d^	0.98 ± 0.02 ^e^	0.01 ± 0.00 ^a^	5
Lactic acid (g L^−1^)	0.52 ± 0.01 ^c^	0.54 ± 0.03 ^c^	0.65 ± 0.02 ^d^	0.39 ± 0.01 ^b^	0.04 ± 0.00 ^a^	0.37 ± 0.01 ^b^	4
Density (g L^−1^)	986 ± 0 ^b^	987 ± 0 ^c^	985 ± 0 ^a^	986 ± 0 ^b^	985 ± 0 ^a^	985 ± 0 ^a^	3
Reducing sugars (g L^−1^)	0.96 ± 0.00 ^c^	1.20 ± 0.00 ^d^	1.1 ± 0.1 ^c,d^	0.96 ± 0.00 ^c^	0.8 ± 0.1 ^b^	0.70 ± 0.00 ^a^	4
TPI	8.75 ± 0.03 ^e^	10.23 ± 0.02 ^f^	7.57 ± 0.01 ^a^	8.44 ± 0.02 ^c^	8.52 ± 0.05 ^d^	8.33 ± 0.02 ^b^	6
Absorbance 420 nm	0.1938 ± 0.0003 ^e^	0.2083 ± 0.003 ^f^	0.079 ± 0.02 ^a^	0.1127 ± 0.0003 ^b^	0.1566 ± 0.0006 ^c^	0.1685 ± 0.0004 ^d^	6

**Table 2:** Mean contents and standard deviations of the major volatile compounds and polyols (mg L^−1^) in wines from three wineries located in the top-quality zone “Sierra Montilla” (Protected Designation of Origin of “Montilla-Moriles”) during the 2021 and 2022 vintages. ^a. b. c. d. e^: Different letters in the same row indicate homogeneous groups (HGs) at the *p* ≤ 0.05 significance level. Identification of wine samples: CN = Cañada Navarro; LS = Lagar Saavedra; LB = Los borbones.

Major Volatiles	CAS	CN2021	CN2022	LS2021	LS2022	LB2021	LB2022	HGs
**Carbonyl Compounds (3)**								
Acetaldehyde	75-07-0	246 ± 16 ^d^	104 ± 4 ^a^	211 ± 6 ^c^	168 ± 8 ^b^	241 ± 1 ^d^	99 ± 5 ^a^	4
1.1-Diethoxyethane	105-57-7	2.4 ± 0.2 ^d^	0 ^a^	0.81 ± 0.04 ^b^	0 ^a^	1.19 ± 0.04 ^c^	0 ^a^	4
Acetoin	513-86-0	122 ± 12 ^c^	50 ± 3 ^b^	56 ± 2 ^b^	46 ± 4 ^b^	50 ± 4 ^b^	33 ± 1 ^a^	3
**Alcohols (6)**								
2-Phenylethanol	60-12-8	22 ± 2 ^a^	46 ± 2 ^b^	64 ± 2 ^d,e^	68 ± 3 ^e^	59 ± 1 ^c^	63 ± 3 ^c,d^	5
Methanol	67-56-1	103 ± 7 ^b,c^	87 ± 7 ^a^	92 ± 8 ^a,b^	89 ± 5 ^a^	105 ± 9 ^c^	84 ± 5 ^a^	3
1-Propanol	71-23-8	28 ± 2 ^c^	51 ± 2 ^e^	22.8 ± 0.8 ^b^	32 ± 1 ^d^	18.3 ± 0.6 ^a^	20.9 ± 0.2 ^b^	5
Isobutanol	78-83-1	37 ± 3 ^d^	41.5 ± 0.6 ^e^	23.9 ± 0.6 ^c^	18.4 ± 0.5 ^a^	17.6 ± 0.7 ^a^	21.1 ± 0.4 ^b^	5
2-Methyl-1-Butanol	137-32-6	30 ± 1 ^a^	50 ± 1 ^c^	51.2 ± 0.5 ^c^	34.4 ± 0.7 ^b^	49.9 ± 0.9 ^c^	50.1 ± 0.3 ^c^	3
3-Methyl-1-Butanol	123-51-3	193 ± 9 ^a^	247 ± 4 ^b^	284 ± 6 ^d^	264 ± 4 ^c^	249 ± 2 ^b^	268 ± 3 ^c^	4
**Esters (3)**								
Ethyl Acetate	141-78-6	61 ± 1 ^d^	66.1 ± 0.8 ^e^	42 ± 1 ^b^	62.3 ± 0.2 ^d^	26.5 ± 0.9 ^a^	46.2 ± 0.6 ^c^	5
Ethyl Lactate	97-64-3	61 ± 4 ^d^	14 ± 1 ^b^	64 ± 3 ^d^	11.8 ± 0.6 ^a,b^	22.8 ± 0.4 ^c^	10.7 ± 0.8 ^a^	4
Diethyl Succinate	123-25-1	10.5 ± 0.5 ^d^	6.4 ± 0.4 ^b^	17.9 ± 0.9 ^e^	8 ± 0.5 ^c^	6.4 ± 0.3 ^b^	5.2 ± 0.2 ^a^	5
**Polyols (3)**								
2.3-Butanediol (*levo*)	24347-58-8	626 ± 59 ^a^	1435 ± 56 ^b^	676 ± 48 ^a^	1426 ± 102 ^b^	629 ± 45 ^a^	648 ± 62 ^a^	2
2.3-Butanediol (*meso*)	5341-95-7	265 ± 13 ^b^	473 ± 19 ^c^	228 ± 15 ^a^	457 ± 32 ^c^	205 ± 13 ^a^	229 ± 11 ^a^	3
Glycerol	56-81-5	8563 ± 830 ^a^	16,108 ± 965 ^c^	9106 ± 809 ^a^	18,225 ± 1319 ^d^	8071 ± 235 ^a^	11,298 ± 1127 ^b^	4

**Table 3:** Mean of the contents and standard deviations of the 46 minor volatile compounds (µg L^−1^ ≡ ppb) in wines from three wineries located in the top-quality zone “Sierra Montilla” (Protected Designation of Origin of “Montilla-Moriles”) during the 2021 and 2022 vintages. ^a, b, c, d, e, f^: Different letters in the same row indicate homogeneous groups (HGs) at the *p* ≤ 0.05 significance level. Identification of wine samples: CN = Cañada Navarro; LS = Lagar Saavedra; LB = Los borbones.

Compounds	CAS	CN2021	CN2022	LB2021	LB2022	LS2021	LS2022	HGs
**Acetates (6)**								
Butyl Acetate	123-86-4	3.45 ± 0.05 ^c^	1.6 ± 0.2 ^a^	2.1 ± 0.1 ^b^	2.14 ± 0.01 ^b^	3.2 ± 0.4 ^c^	1.5 ± 0.2 ^a^	3
Isoamyl Acetate	123-92-2	1893 ± 165 ^c^	2501 ± 162 ^d^	708 ± 5 ^b^	3509 ± 114 ^e^	387 ± 24 ^a^	1763 ± 49 ^c^	5
Hexyl Acetate	142-92-7	21 ± 1 ^b^	98 ± 4 ^d^	7.7 ± 0.1 ^a^	70.4 ± 0.1 ^c^	2.6 ± 0.7 ^a^	76 ± 6 ^c^	4
Octyl Acetate	112-14-1	2.74 ± 0.08 ^a^	2.9 ± 0.1 ^a,b^	3.2 ± 0.3 ^a,b^	4.9 ± 0.2 ^d^	3.6 ± 0.9 ^b^	2.76 ± 0.04 ^a^	3
Ethyl-phenyl Acetate	101-97-3	2.3 ± 0.1 ^a^	1.8 ± 0.1 ^a^	3.41 ± 0.03 ^a^	96 ± 5 ^d^	9.5 ± 0.6 ^b^	81 ±4 ^c^	4
2-Phenyl-ethyl Acetate	103-45-7	940 ± 32 ^a^	3603 ± 110 ^b^	1088 ± 77 ^a^	3573 ± 177 ^b^	872 ± 79 ^a^	3374 ± 224 ^b^	2
**Ethyl Esters (12)**								
Ethyl Isobutyrate	97-62-1	93 ± 8 ^d^	11.2 ± 0.9 ^b^	12.7 ± 0.9 ^b^	14 ± 4 ^b^	79.6 ± 0.4 ^c^	0 ^a^	4
Ethyl Butyrate	105-54-4	116.17 ± 0.09 ^d^	100 ± 5 ^c^	62 ± 6 ^a^	115 ± 6 ^d^	85 ± 4 ^b^	60 ± 2 ^a^	4
Ethyl 2-methyl-butyrate	7452-79-1	9.8 ± 0.7 ^c^	0 ^a^	8.0 ± 0.5 ^b^	0 ^a^	24.3 ± 0.9 ^d^	0 ^a^	4
Ethyl 3-methyl-butyrate	108-64-5	16 ± 1 ^c^	0 ^a^	12.2 ± 0.6 ^b^	0 ^a^	44 ± 2 ^d^	0 ^a^	4
Ethyl Hexanoate	123-66-0	601 ± 36 ^d^	422 ± 25 ^c^	14 ± 1 ^a^	213 ± 11 ^b^	2.6 ± 0.7 ^a^	184 ± 7 ^b^	4
Ethyl Heptanoate	106-30-9	0.86 ± 0.05 ^d^	0.30 ± 0.01 ^c^	0.13 ± 0.00 ^b^	0 ^a^	0 ^a^	0 ^a^	4
Ethyl Octanoate	106-32-1	590 ± 28 ^d^	772 ± 55 ^e^	0 ^a^	277 ± 10 ^c^	94 ± 9 ^b^	262 ± 18 ^c^	5
Ethyl Decanoate	110-38-3	443 ± 11 ^b^	1988 ± 104 ^d^	45 ± 1 ^a^	847 ± 20 ^c^	58 ± 5 ^a^	811 ± 12 ^c^	4
Ethyl Benzoate	93-89-0	1.5 ± 0.1 ^c^	2.71 ± 0.09 ^d^	0 ^a^	0 ^a^	0.78 ± 0.09 ^b^	0 ^a^	4
Ethyl Dodecanoate	106-33-2	20 ± 2 ^a^	555 ± 42 ^d^	10.9 ± 0.3 ^a^	161 ± 10 ^c^	10.2 ± 0.7 ^a^	59 ± 4 ^b^	4
Ethyl Tetradecanoate	124-06-1	10.8 ± 0.5 ^b^	18 ± 2 ^c^	6.2 ± 0.3 ^a^	19.2 ± 0.3 ^d^	6.0 ± 0.3 ^a^	12 ± 1 ^b^	4
Ethyl Hexadecanoate	628-97-7	23 ± 2 ^c^	61 ± 1 ^d^	14 ± 1 ^b^	60 ± 1 ^d^	6.2 ± 0.3 ^a^	24 ± 1 ^c^	4
**Other Esters (3)**								
Phenethyl Butyrate	103-52-6	0 ^a^	2.12 ± 0.01 ^c^	0 ^a^	2.0 ± 0.1 ^c^	1 ± 0.1 ^b^	2.5 ± 0.1 ^d^	4
Phenethyl Hexanoate	6290-37-5	0 ^a^	0 ^a^	0 ^a^	0 ^a^	0 ^a^	0.44 ± 0.02 ^b^	2
Phenethyl Benzoate	94-47-3	156 ± 9 ^b^	3.16 ± 0.08 ^a^	3.20 ± 0.02 ^a^	3.3 ± 0.2 ^a^	3.2 ± 0.3 ^a^	3.1 ± 0.2 ^a^	3
**Higher Alcohols (5)**								
Hexanol	111-27-3	885 ± 41 ^a^	1016 ± 98 ^a,b^	1564 ± 141 ^c^	1644 ± 59 ^c^	1055 ± 57 ^b^	1548 ± 30 ^c^	3
2-Ethyl-1-Hexanol	104-76-7	453 ± 34 ^c^	32 ± 4 ^a^	45 ± 3 ^a,b^	51 ± 1 ^ab^	59 ± 2 ^b^	28 ± 8 ^a^	3
Furanmethanol	98-00-0	0 ^a^	2.87 ± 0.04 ^b^	6.8 ± 0.4 ^c^	6.7 ± 0.4 ^c^	3.50 ± 0.08 ^b^	2.5 ± 0.1 ^a,b^	3
Octanol	111-87-5	97 ± 3 ^c^	0 ^a^	0 ^a^	0 ^a^	0 ^a^	88 ± 4 ^b^	3
Dodecanol	112-53-8	7.9 ± 0.6 ^b^	10.9 ± 0.6 ^c^	6.0 ± 0.3 ^a^	10.30 ± 0.01 ^c^	8.2 ± 0.2 ^b^	9 ± 2 ^b^	3
**Phenols (2)**								
4-Ethyl Guaiacol	2785-89-9	319 ± 13 ^c^	0 ^a^	0 ^a^	0 ^a^	219 ± 7 ^b^	0 ^a^	2
2-Methoxy-4-Vinyl-phenol	7786-61-0	0 ^a^	357 ± 5 ^e^	39 ± 2 ^b^	120 ± 11 ^d^	0 ^a^	50 ± 4 ^c^	5
**Lactones (3)**								
γ-Butyrolactone	96-48-0	11,924 ± 596 ^a^	12,738 ± 1015 ^a,b^	15,723 ± 1392 ^d^	14,002 ± 174 ^b,c^	13,610 ± 795 ^a,b,c^	14,559 ± 1185 ^c,d^	4
γ-Nonalactone	104-61-0	15.2 ± 0.7 ^b,c^	22.8 ± 0.8 ^e^	13.46 ± 0.01 ^a,b^	16 ± 2 ^cd^	11.3 ± 0.2 ^a^	18 ± 2 ^d^	5
β-Damascenone	23726-93-4	13.0 ± 0.4 ^b^	63 ± 3 ^d^	4.9 ± 0.3 ^a^	27.3 ± 0.3 ^c^	5.7 ± 0.3 ^a^	25.0 ± 0.4 ^c^	4
**Carbonyl Compounds (8)**								
Hexanal	66-25-1	3.6 ± 0.2 ^a^	3.4 ± 0.5 ^a^	4.9 ± 0.4 ^b^	5.9 ± 0.9 ^b,c^	6.1 ± 0.9 ^c^	3.4 ± 0.5 ^a^	3
Furfural	98-01-1	419 ± 28 ^a^	438 ± 9 ^a^	876 ± 36 ^c^	434 ± 12 ^a^	758 ± 109 ^b^	403 ± 21 ^a^	3
Benzaldehyde	100-52-7	0 ^a^	0.001 ± 0.000 ^a^	2.3 ± 0.3 ^b^	0 ^a^	3.0 ± 0.6 ^c^	6.0 ± 0.1 ^d^	4
Octanal	124-13-0	0 ^a^	2.0 ± 0.2 ^d^	1 ± 0.1 ^b^	2.32 ± 0.06 ^e^	1.3 ± 0.1 ^c^	2.2 ± 0.1 ^e^	5
Decanal	112-31-2	5.6 ± 0.2 ^a^	9.8 ± 0.9 ^b^	6.6 ± 0.6 ^a^	10 ± 1 ^b^	7 ± 2 ^a^	11.6 ± 0.8 ^b^	2
(E)-2-Nonenal	18829-56-6	6.4 ± 0.4 ^c^	0 ^a^	4.3 ± 0.3 ^b^	0 ^a^	4.3 ± 0.5 ^b^	0 ^a^	3
Phenylacetaldehyde	122-78-1	0 ^a^	0 ^a^	0 ^a^	0 ^a^	9.4 ± 0.9 ^b^	0 ^a^	2
3-Heptanone	106-35-4	1.6 ± 0.3 ^c^	0.001 ± 0.000 ^a^	0.001 ± 0.000 ^a^	0.001 ± 0.000 ^a^	0 ^a^	0.4 ± 0.1 ^b^	3
**Terpenes and Derivatives (7)**								
(DL)-Limonene	138-86-3	0 ^a^	5217 ± 213 ^b^	0 ^a^	28 ± 3 ^a^	0 ^a^	5348 ± 55 ^b^	2
(E)-Geranyl Acetone	3796-70-1	1.2 ± 0.1 ^a^	1.6 ± 0.1 ^b^	1.3 ± 0.2 ^a^	1.16 ± 0.08 ^a^	1.76 ± 0.03 ^b^	2.3 ± 0.1 ^c^	3
Geranyl Acetate	105-87-3	3.08 ± 0.06 ^a^	5.8 ± 0.3 ^b^	7.9 ± 0.4 ^c^	38.7 ± 0.5 ^f^	8.9 ± 0.5 ^d^	35.5 ± 0.5 ^e^	6
(E)-Methyldihydrojasmonate	2630-39-9	0.7 ± 0.2 ^a^	2.3 ± 0.2 ^c^	1.7 ± 0.5 ^b^	1.3 ± 0.1 ^b^	0.8 ± 0.1 ^a^	1.4 ± 0.2 ^b^	3
(Z)-Geranyl Acetone	3879-26-3	1.93 ± 0.05 ^a,b^	1.98 ± 0.03 ^a,b^	1.90 ± 0.08 ^a^	1.88 ± 0.08 ^a^	1.83 ± 0.04 ^a^	1.89 ± 0.04 ^a,b^	2
(Z)-Citral	106-26-3	0 ^a^	0 ^a^	22.9 ± 0.5 ^b^	0 ^a^	0 ^a^	0 ^a^	2
(Z)-Nerolidol	7212-44-4	0 ^a^	0.95 ± 0.05 ^c^	0.30 ± 0.02 ^b^	0.98 ± 0.03 ^c^	0 ^a^	0 ^a^	3

## Data Availability

Data are available under petition to the authors.

## Supplementary Materials

The following supporting information can be downloaded at: https://www.mdpi.com/article/10.3390/molecules30142982/s1, Table S1: Multiple analysis of variance (MANOVA) with terroir, vintage, and interaction as variation factors. List of the compounds affected not significantly at *p* ≤ 0.01 values. Table S2: Principal component analysis of the major volatile compounds and polyols. Variance explained and loads of the compounds in the first three principal components. Table S3: Principal component analysis of 46 minor volatiles quantified. Variance explained and component loads. Table S4: Principal component analysis of seven terpenic compounds quantified. Variance explained and component loads. Table S5: Principal component analysis of 21 esters quantified. Variance explained and component loads. Figure S1: Heat map of 46 minor and 14 major volatile compounds in wines obtained in three terroirs and two vintages. Figure S2: Principal component analysis. Sample scores of the two first principal components performed with the following: A: the 46 minor volatiles quantified; B: the seven terpenic compounds quantified.
