# Supplementary material for: Volatilome Analysis for Differentiating Terroir Expression: A Case Study of Three Wineries in a Limestone-Rich, Warm-Climate Region
Source: Molecules. 2025 Jul 16;30(14):2982. doi: 10.3390/molecules30142982 (PMC12299196; doi:10.3390/molecules30142982)
Supplement: Supplementary file 1 [file molecules-30-02982-s001.zip › molecules-3682067-supplementary.pdf]

**Supplementary Material**

**Table S1**: Multiple analysis of variance with terroir, vintage, and their interaction as variation factors. List of the compounds not significantly affected at $p \leq 0.01$ values by some of these factors. All remaining compounds quantified are dependent on terroir, vintage, and their interaction at $p \leq 0.01$ values.

| Compounds | Terroir | Vintage | Interaction |
|---|---|---|---|
| GENERAL PARAMETERS | | | |
| Ethanol | 0.0000 | 0.0111 | 0.0000 |
| Reducing sugars | 0.0000 | 0.9236 | 0.0011 |
| MAJOR VOLATILES | | | |
| Methanol | 0.5304 | 0.0014 | 0.0920 |
| Isobutanol | 0.0000 | 0.2129 | 0.0000 |
| 2-Methyl-1-butanol | 0.0000 | 0.0149 | 0.0000 |
| MINOR VOLATILES | | | |
| 2-Phenylethylacetate | 0.0000 | 0.0515 | 0.4645 |
| Ethylbutyrate | 0.0000 | 0.1352 | 0.0000 |
| 2-Furanmethanol | 0.0002 | 0.4445 | 0.1212 |
| Dodecanol | 0.0641 | 0.0000 | 0.0063 |
| Benzaldehyde | 0.0000 | 0.1340 | 0.0000 |
| Decanal | 0.1105 | 0.0000 | 0.7311 |
| (E)-Geranylacetone | 0.0360 | 0.2712 | 0.3779 |
| | | | |

## Supplementary Material

**Figure S1**. Heat map of 46 minor and 14 major volatile compounds in wines obtained in three terroirs and two vintages.

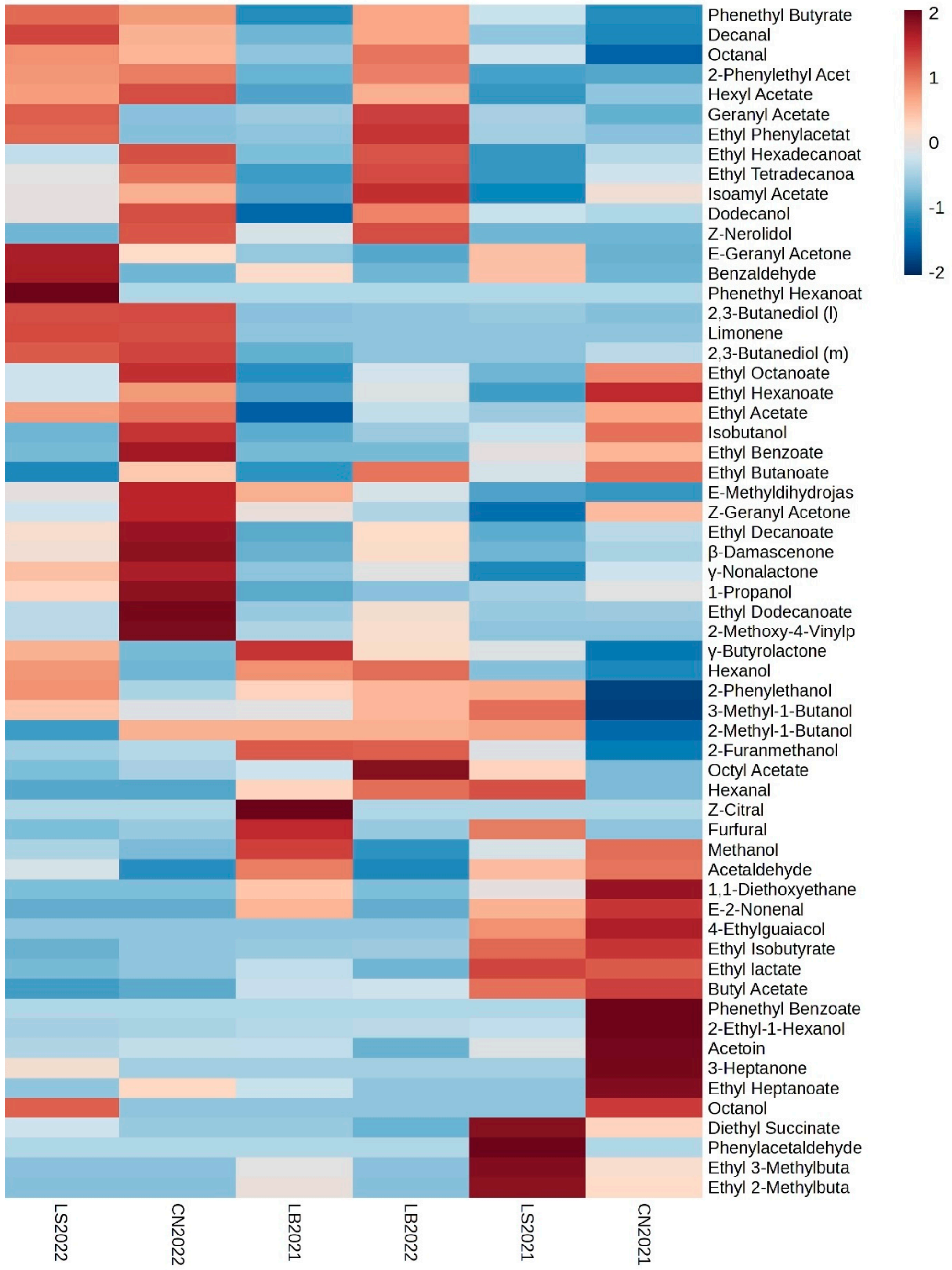

**Supplementary Material**

**Table S2**: Principal component analysis of the major volatile compounds and polyols. Variance explained and loads of the compounds in the first three principal components.

| | *Component 1* | *Component 2* | *Component 3* |
|---|---|---|---|
| *Variance explained* | *48.45 %* | *24.67 %* | *19.66 %* |
| *Cumulative variance* | *48.45 %* | *73.12 %* | *92.78 %* |
| Acetaldehyde | **0.274149** | -0.0100892 | -0.381384 |
| Ethyl acetate | -0.0997195 | -0.206758 | 0.469443 |
| 1.1-Diethoxyethane | 0.171809 | **0.230945** | -0.424986 |
| Methanol | **0.29864** | **0.209674** | -0.189618 |
| 1-Propanol | **0.242047** | -0.213212 | 0.331429 |
| Isobutanol | 0.0571141 | **0.489621** | 0.0981173 |
| 2-Methyl-1-butanol | 0.0972469 | **0.452344** | 0.228024 |
| 3-Methyl-1-butanol | **0.207677** | **0.313807** | 0.295899 |
| Acetoin | **0.270323** | 0.192426 | 0.269596 |
| Ethyl lactate | **0.308539** | -0.245427 | -0.148264 |
| 2.3-Butanediol (levo) | **0.296141** | -0.26503 | 0.132127 |
| 2.3-Butanediol (meso) | **0.328711** | -0.187991 | 0.0695944 |
| Diethyl succinate | **0.355419** | 0.0338827 | -0.0779837 |
| 2-Phenylethanol | **0.286825** | -0.253524 | -0.0378767 |
| Glycerol | **0.330374** | 0.0575054 | 0.184653 |

This table shows the contribution of each variable to the equations of the principal components. For example, the first principal component follows the equation:

**PC1**= 0.274149*Acetaldehyde - 0.0997195*Ethyl acetate + 0.171809*1.1-Diethoxyethane + 0.29864*Methanol + 0.242047*1-Propanol + 0.0571141*Isobutanol + 0.0972469*2-Methyl-1-butanol + 0.207677*3-Methyl-1-butanol + 0.270323*Acetoin + 0.308539*Ethyl lactate + 0.296141*2.3-Butanediol (levo) + 0.328711*2.3-Butanediol (meso) + 0.355419*Diethyl succinate + 0.286825*2-Phenyl-ethanol + 0.330374*Glycerol

**Supplementary Material**

**Figure S2**. Principal component analysis. Sample scores of the two first principal components performed with the following: **A:** the 46 minor volatiles quantified; **B**: the 7 terpenic compounds quantified.

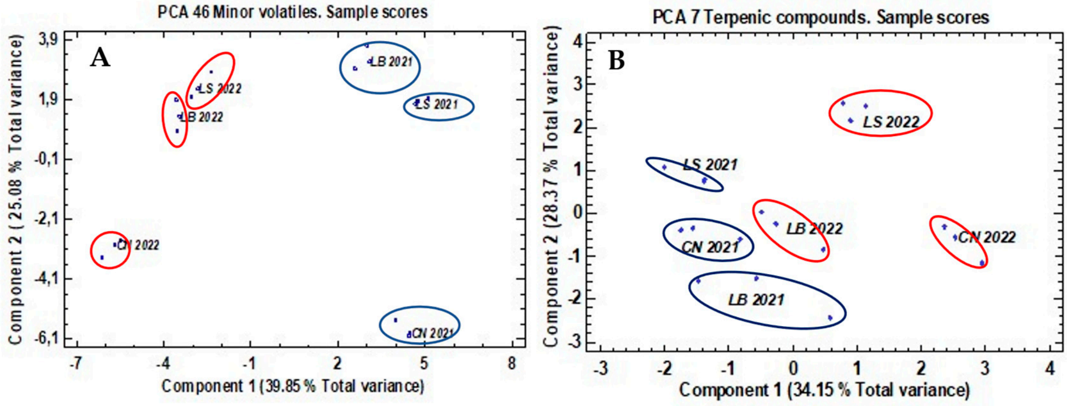

**Supplementary Material**

**Table S3**: Principal component analysis of the 46 minor volatiles quantified. Variance explained and component loads.

This procedure involves performing a principal component analysis that allows us to obtain a reduced number of linear combinations of the 46 variables explaining the greatest variability in the data. In this case, three components have been extracted and together, they explain 77.595% of the total variability in the original data.

| | *Component 1* | *Component 2* | *Component 3* |
|---|---|---|---|
| *Variance explained* | *39.853 %* | *25.082 %* | *12.660 %* |
| *Cumulative variance* | *39.853 %* | *64.934 %* | *77.595 %* |
| Ethyl Isobutyrate | 0.180485 | -0.147592 | 0.0309146 |
| Ethyl Butanoate | -0.0201646 | -0.203867 | 0.201503 |
| Hexanal | 0.073215 | 0.145504 | 0.243787 |
| Butyl Acetate | 0.189158 | -0.112464 | 0.074554 |
| Furfural | 0.138329 | 0.1597 | 0.143941 |
| 2-Furanmethanol | -0.0360614 | 0.197719 | 0.218989 |
| Ethyl 2-methylbutanoate | 0.196038 | 0.024818 | 0.0701786 |
| Ethyl 3-methylbutanoate | 0.189649 | 0.0267927 | 0.0689326 |
| Hexanol | -0.0739631 | 0.226306 | -0.0144526 |
| Isoamyl Acetate | -0.173838 | -0.0951522 | 0.0753763 |
| 3-Heptanone | 0.0909879 | -0.210314 | -0.18081 |
| γ-Butyrolactone | 0.00304227 | 0.238808 | -0.0117391 |
| Benzaldehyde | 0.0211684 | 0.18524 | -0.30379 |
| Ethyl Hexanoate | -0.0456611 | -0.278776 | -0.0577879 |
| Octanal | -0.18719 | 0.145081 | 0.00878431 |
| Hexyl Acetate | -0.227359 | -0.0475155 | -0.0562947 |
| 2-ethyl-1-hexanol | 0.117862 | -0.226655 | -0.0632561 |
| Limonene | -0.168483 | -0.0194717 | -0.220653 |
| Octanol | 0.0383313 | -0.121427 | -0.344871 |
| Ethyl Heptanoate | 0.0714191 | -0.265083 | -0.0370111 |
| Ethyl Octanoate | -0.109654 | -0.255113 | -0.0126742 |
| Phenylacetaldehyde | 0.121384 | 0.074094 | 0.0720027 |
| E-2-Nonenal | 0.218446 | -0.0864554 | 0.0225369 |
| Ethyl Benzoate | -0.0528234 | -0.226841 | 0.0740162 |
| Decanal | -0.186155 | 0.0837135 | -0.112348 |
| Octyl Acetate | -0.042891 | 0.096332 | 0.250931 |
| Z-Citral | 0.072685 | 0.131134 | 0.0859467 |
| Ethyl Phenylacetate | -0.117797 | 0.123943 | -0.0924029 |
| 2-Phenylethyl Acetate | -0.228002 | 0.01797 | -0.0328045 |
| 4-ethylguaiacol | 0.175109 | -0.165465 | -0.0298536 |
| 2-methoxy-4-vinylphenol | -0.188734 | -0.0818706 | 0.128621 |
| γ-Nonalactone | -0.194147 | -0.115216 | -0.0549007 |
| Geranyl Acetate | -0.120718 | 0.145767 | -0.0989338 |
| β-Damascenone | -0.203208 | -0.109619 | 0.0380853 |
| Ethyl Decanoate | -0.204055 | -0.120765 | 0.0174278 |
| E-Geranyl Acetone | -0.0566344 | 0.0928777 | -0.292944 |
| Phenethyl Butyrate | -0.198754 | 0.0579066 | -0.0903878 |
| Z-Geranyl Acetone | -0.086558 | -0.139462 | 0.00393167 |
| Dodecanol | -0.170593 | -0.0895469 | 0.0780057 |
| Ethyl Dodecanoate | -0.18098 | -0.107629 | 0.121567 |
| Phenethyl Hexanoate | -0.0689462 | 0.0938398 | -0.369842 |
| E-Methyldihydrojasmonate | -0.156604 | 0.015821 | 0.0812812 |
| Z-Nerolidol | -0.174464 | -0.0201574 | 0.268296 |

| | | | |
|---|---|---|---|
| Ethyl Tetradecanoate | -0.19697 | -0.0919306 | 0.0925829 |
| Phenethyl Benzoate | 0.108235 | -0.231619 | -0.0815186 |
| Ethyl Hexadecanoate | -0.200697 | -0.0824868 | 0.152127 |

This table shows the equations of the three principal components. For example, the first principal component has the following equation:

**PC1** = 0.180485*Ethyl Isobutyrate - 0.0201646*Ethyl Butanoate + 0.073215*Hexanal + 0.189158*Butyl Acetate + 0.138329*Furfural - 0.0360614*2-Furanmethanol + 0.196038*Ethyl 2-methylbutanoate + 0.189649*Ethyl 3-methylbutanoate - 0.0739631*Hexanol - 0.173838*Isoamyl Acetate + 0.0909879*3-Heptanone + 0.00304227*Î³-Butyrolactone + 0.0211684*Benzaldehyde - 0.0456611*Ethyl Hexanoate - 0.18719*Octanal - 0.227359*Hexyl Acetate + 0.117862*2-ethyl-1-hexanol - 0.168483*Limonene + 0.0383313*Octanol + 0.0714191*Ethyl Heptanoate - 0.109654*Ethyl Octanoate + 0.121384*Phenylacetaldehyde + 0.218446*E-2-Nonenal - 0.0528234*Ethyl Benzoate - 0.186155*Decanal - 0.042891*Octyl Acetate + 0.072685*Z-Citral - 0.117797*Ethyl Phenylacetate - 0.228002*2-Phenylethyl Acetate + 0.175109*4-ethylguaiacol - 0.188734*2-methoxy-4-vinylphenol - 0.194147*Î³-Nonalactone - 0.120718*Geranyl Acetate - 0.203208*Î²-Damascenone - 0.204055*Ethyl Decanoate - 0.0566344*E-Geranyl Acetone - 0.198754*Phenethyl Butyrate - 0.086558*Z-Geranyl Acetone - 0.170593*Dodecanol - 0.18098*Ethyl Dodecanoate - 0.0689462*Phenethyl Hexanoate - 0.156604*E-Methyldihydrojasmonate - 0.174464*Z-Nerolidol - 0.19697*Ethyl Tetradecanoate + 0.108235*Phenethyl Benzoate - 0.200697*Ethyl Hexadecanoate

In this equation, the values of the variables have been standardized by subtracting their mean values and dividing them by their standard deviations.

## Supplementary Material

**Table S4**: Principal component analysis of the seven terpenic compounds quantified. Variance explained and component loads.

This procedure involves performing a principal component analysis that allows us to obtain a reduced number of linear combinations of the seven variables explaining the greatest variability in the data. In this case, three components have been extracted and together, they explain 80.109% of the total variability in the original data.

| | *Component 1* | *Component 2* | *Component 3* |
|---|---|---|---|
| *Variance explained* | *34.150 %* | *28.367 %* | *17.591 %* |
| *Cumulative variance* | *34.150 %* | *62.517 %* | *80.109 %* |
| Limonene | 0.535859 | 0.326601 | -0.186023 |
| Z-Citral | -0.0956606 | -0.423874 | -0.328509 |
| Geranyl Acetate | 0.111704 | 0.35715 | 0.599398 |
| E-Geranyl Acetone | 0.229833 | 0.578914 | -0.339671 |
| Z-Geranyl Acetone | 0.429922 | -0.298824 | -0.190022 |
| E-Methyldihydrojasmonate | 0.561866 | -0.260265 | -0.0971437 |
| Z-Nerolidol | 0.371318 | -0.306483 | 0.580756 |

This table shows the equations of the three principal components. For example, the first principal component has the following equation:

**PC1** = 0.535859*Limonene - 0.0956606*Z-Citral + 0.111704*Geranyl Acetate + 0.229833*E-Geranyl Acetone + 0.429922*Z-Geranyl Acetone + 0.561866*E-Methyldihydrojasmonate + 0.371318*Z-Nerolidol

In this equation, the values of the variables have been standardized by subtracting their mean values and dividing them by their standard deviations.

## Supplementary Material

**Table S5**: Principal component analysis of the 21 esters quantified. Variance explained and component loads.

This procedure involves performing a principal component analysis that allows us to obtain a reduced number of linear combinations of the 21 variables explaining the greatest variability in the data. In this case, three components have been extracted and together, they explain 85.581 % of the total variability in the original data.

| | *Component 1* | *Component 2* | *Component 3* |
|---|---|---|---|
| *Variance explained* | *46.805* | *27.137* | *11.639* |
| *Cumulative variance* | *46.805* | *73.942* | *85.581* |
| Ethyl Isobutyrate | **0.22007** | -0.252776 | 0.127864 |
| Ethyl Butanoate | -0.0726987 | -0.311271 | 0.391871 |
| Butyl Acetate | **0.233552** | -0.2119 | 0.237573 |
| Ethyl 2-methylbutanoate | **0.279885** | -0.0287245 | 0.0671577 |
| Ethyl 3-methylbutanoate | **0.272033** | -0.0238038 | 0.0723062 |
| Isoamyl Acetate | -0.268756 | -0.0961841 | 0.272895 |
| Ethyl Hexanoate | -0.11103 | -0.362191 | -0.0572409 |
| Hexyl Acetate | -0.312288 | 0.00113907 | -0.0804259 |
| Ethyl Heptanoate | 0.050535 | -0.377821 | -0.0939309 |
| Ethyl Octanoate | -0.180835 | -0.326249 | -0.137995 |
| Ethyl Benzoate | -0.0851565 | -0.323288 | -0.224833 |
| Octyl Acetate | -0.0574852 | 0.106694 | 0.575055 |
| Ethyl Phenylacetate | -0.163207 | **0.227621** | 0.283644 |
| 2-Phenylethyl Acetate | -0.307792 | 0.0878632 | 0.0183283 |
| Ethyl Decanoate | -0.283144 | -0.124736 | -0.149722 |
| Phenethyl Butyrate | -0.255726 | **0.148641** | -0.012525 |
| Ethyl Dodecanoate | -0.24401 | -0.133883 | -0.137045 |
| Phenethyl Hexanoate | -0.0787119 | **0.218868** | -0.278592 |
| Ethyl Tetradecanoate | -0.291548 | -0.0915553 | 0.198154 |
| Phenethyl Benzoate | 0.0972668 | -0.328006 | 0.0203543 |
| Ethyl Hexadecanoate | -0.290467 | -0.0917517 | 0.176498 |
| | | | |

This table shows the equations of the three principal components. For example, the first principal component has the following equation:
**PC1** = 0.22007*Ethyl Isobutyrate - 0.0726987*Ethyl Butanoate + 0.233552*Butyl Acetate + 0.279885*Ethyl 2-methylbutanoate + 0.272033*Ethyl 3-methylbutanoate - 0.268756*Isoamyl Acetate - 0.11103*Ethyl Hexanoate - 0.312288*Hexyl Acetate + 0.050535*Ethyl Heptanoate - 0.180835*Ethyl Octanoate - 0.0851565*Ethyl Benzoate - 0.0574852*Octyl Acetate - 0.163207*Ethyl Phenylacetate - 0.307792*2-Phenylethyl Acetate - 0.283144*Ethyl Decanoate - 0.255726*Phenethyl Butyrate - 0.24401*Ethyl Dodecanoate - 0.0787119*Phenethyl Hexanoate - 0.291548*Ethyl Tetradecanoate + 0.0972668*Phenethyl Benzoate - 0.290467*Ethyl Hexadecanoate

In this equation, the values of the variables have been standardized by subtracting their mean values and dividing them by their standard deviations.
